# Cooperation effects of radiation and ferroptosis on tumor suppression and radiation injury

**DOI:** 10.3389/fcell.2022.951116

**Published:** 2022-09-13

**Authors:** Jing Su, Chenbin Bian, Zhuangzhuang Zheng, Huanhuan Wang, Lingbin Meng, Ying Xin, Xin Jiang

**Affiliations:** ^1^ Jilin Provincial Key Laboratory of Radiation Oncology & Therapy, The First Hospital of Jilin University, Changchun, China; ^2^ Department of Radiation Oncology, The First Hospital of Jilin University, Changchun, China; ^3^ NHC Key Laboratory of Radiobiology, School of Public Health, Jilin University, Changchun, China; ^4^ Department of Hematology and Medical Oncology, Moffitt Cancer Center, Tampa, FL, United States; ^5^ Key Laboratory of Pathobiology, Ministry of Education, Jilin University, Changchun, China

**Keywords:** ferroptosis, oxidative stress, reactive oxygen species (ROS), GPX4, SLC7A11, radiotherapy

## Abstract

Ferroptosis is a kind of oxidative stress-dependent cell death characterized by iron accumulation and lipid peroxidation. It can work in conjunction with radiation to increase reactive oxygen species (ROS) generation and disrupt the antioxidant system, suppressing tumor progression. Radiation can induce ferroptosis by creating ROS, depleting glutathione, activating genes linked to DNA damage and increasing the expression of acyl-CoA synthetase long-chain family member 4 (ACSL4) in tumor cells. Furthermore, ferroptosis can enhance radiosensitivity by causing an iron overload, destruction of the antioxidant system, and lipid peroxidation. Radiation can also cause ferroptosis in normal cells, resulting in radiation injury. The role of ferroptosis in radiation-induced lung, intestinal, skin, and hematological injuries have been studied. In this review, we summarize the potential mechanisms linking ferroptosis, oxidative stress and radiation; analyze the function of ferroptosis in tumor suppression and radiation injury; and discuss the potential of ferroptosis regulation to improve radiotherapy efficacy and reduce adverse effects.

## 1 Introduction

Cancer is one of the most common causes of death worldwide. About 19.3 million new cancer cases and 10.0 million cancer deaths occurred in 2020 (Sung et al., 2021). Radiotherapy is an effective strategy to treat cancer. Ionizing radiation (IR) can directly damage cellular DNA and produce double-strand breaks, resulting in death-related events such as cell cycle arrest, autophagy, and apoptosis (Delaney et al., 2005). Furthermore, radiation can ionize the cytoplasm and mitochondria, resulting in a large amount of reactive oxygen species (ROS) that interact with biological macromolecules and cause permanent damage and cell death. However, radiotherapy frequently fails to achieve the desired effect in various tumor types (Taylor et al., 2018; Pilié et al., 2019; Huang and Zhou, 2020). Radiation resistance leads to poor prognosis and ultimately to tumor relapse and metastasis in patients with cancer (Rycaj and Tang, 2014). Therefore, safe and effective strategies must be identified to enhance radiosensitivity. Radiotherapy (RT) is a double-edged sword. Despite the continuous advancements in precision radiotherapy technology, radiation-induced damage is inevitable (De Ruysscher et al., 2019; Wei et al., 2019). Prevention of radiation damage is an urgent and serious issue.

Ferroptosis was first introduced in 2012 as a highly iron-dependent form of non-apoptotic cell death (Dixon et al., 2012). Ferroptosis differs from apoptosis, necrosis, autophagy, and pyroptosis in terms of morphology, metabolic response, and gene expression (Yagoda et al., 2007; Li et al., 2020; Bertheloot et al., 2021) (Table 1). Morphologically, ferroptosis is characterized by evident mitochondrial contraction, increased membrane density, and loss of mitochondrial crest rather than chromatin aggregation (such as apoptosis) or autophagosome production (such as autophagy) (Bertheloot et al., 2021). At the point of biochemical reaction, ferroptosis is mainly related to the peroxidation of polyunsaturated fatty acid phospholipids (PUFA-PLs). Glutathione peroxidase 4 (GPX4) is a crucial factor in ferroptosis. Inhibition of its activity results in abnormal cell metabolism, weakened antioxidant capacity, and excessive lipid ROS, leading to cell death (Li et al., 2020). Furthermore, the tumor suppressor genes P53 and BAP1 can regulate ferroptosis, but the regulatory mechanisms and effects require further investigation (Jiang et al., 2015; Zhang et al., 2018).

**TABLE 1:** The difference between ferroptosis, apoptosis, autophagy, necroptosis, and pyroptosis.

	Ferroptosis	Apoptosis	Autophagy	Necroptosis	Pyroptosis
Morphology	Obvious mitochondrial contraction, increased membrane density	Cell shrinkage, chromatincon densation,	Formation of autophagosomes	Swelling of the cytoplasm and organelles, rupture of the cell membrane	Swelling of cells, rupture of the cell membrane
	reduced or disappeared mitochondrial cristae	formation of apoptotic bodies and disintegration of the cytoskeleton			
	the nuclear volume doesn’t change	no significant changes in mitochondrial structure			
Biochemical reaction	Iron accumulation and plasma membrane lipid peroxidation	DNA strand breaks	Lysosomal activity is enhanced	Metabolic functions such as ion gradient and ATP production are irreversibly lost	Cell contents and pro-inflammatory cytokines release and inflammasome sensors active
Gene expression	GPX4, SLC7A11, NRF2, ATF4, P53, HSPB1, ACSL4, FSP1, DHODH, TFR1	Caspase, Bcl-2, Bax, P53, Fas	ATG5, ATG7, Beclin-1, DRAM3, TFEB, MAPLC3	RIP1, RIP3	GSDMA, GSDMC, GSDMD, GSDME

Recent studies have demonstrated that ferroptosis plays a crucial role in radiotherapy-induced cell death (Chen et al., 2021; Lei et al., 2021). However, further research into the exact mechanisms of ferroptosis and radiation is required. In this study, the role and mechanism of ferroptosis in radiation were examined to investigate treatment options that may improve radiotherapy efficacy and reduce radiation damage.

## 2 The role of ferroptosis in tumors

**FIGURE 1:**
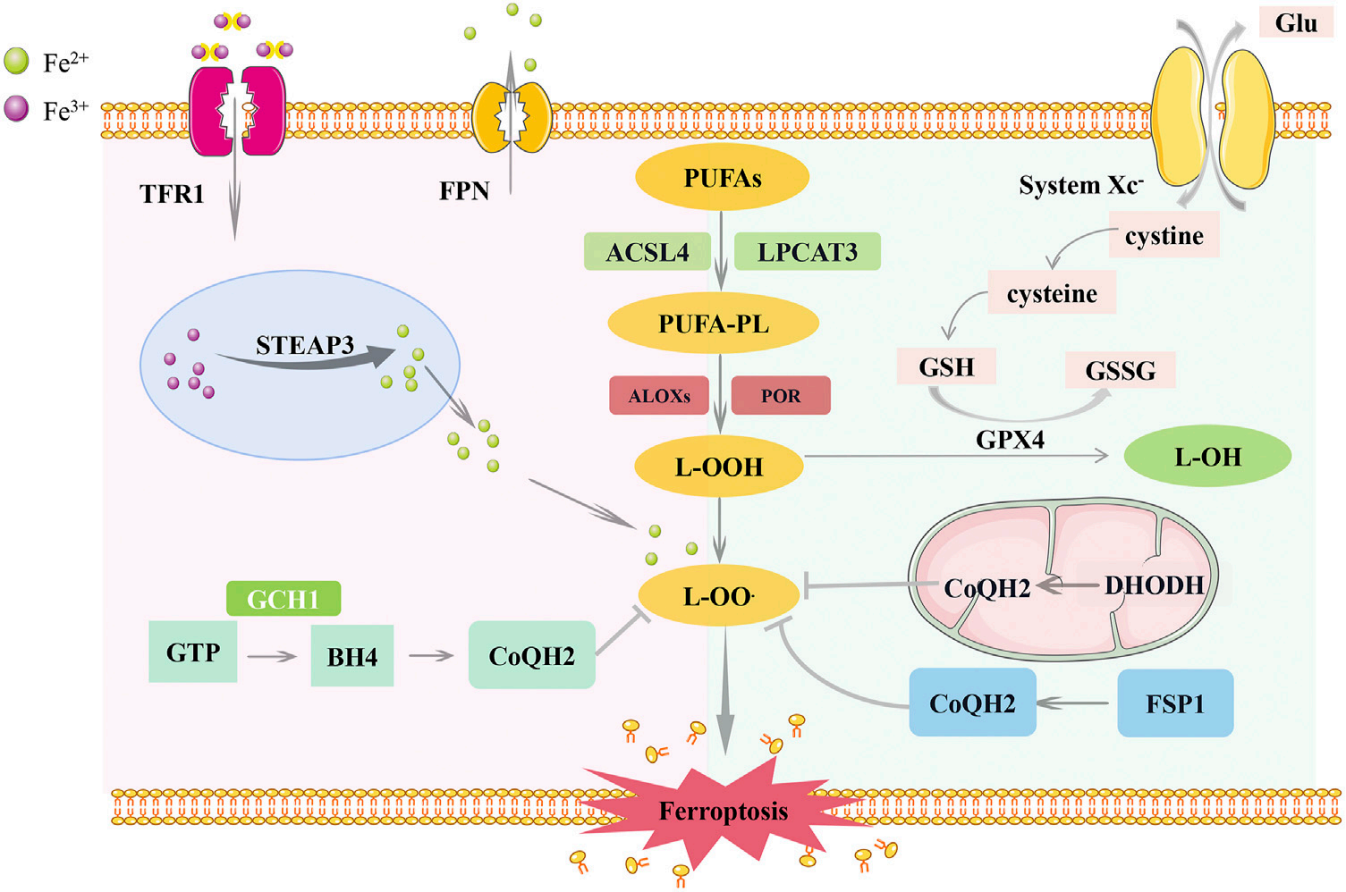
Regulatory pathways of ferroptosis. Ferroptosis is co-regulated by iron metabolism, lipid metabolism, and antioxidant systems. The inhibition of lipid peroxidation is mainly mediated by the SLC7A11-GSH-GPX4 pathway, FSP1-CoQ10-NAD (P)H pathway, GCH1-BH4-DHFR pathway and DHO-DHODH-OA pathway. Among them, the FSP1 pathway exists in the cytoplasm, the DHODH pathway exists in the mitochondria, and the GPX4 pathway plays a role in both. *SLC7A11, Cystine/glutamate antiporter solute carrier family seven member 11; GPX4, glutathione peroxidase 4; GSH, glutathione; FSP1, ferroptosis suppressor protein 1; GGH1, GTP Cyclohydrolase 1; BH4, tetrahydrobiopterin; DHFR, dihydrofolate reductase; DHO, dehydrogenation of dihydroorotate; DHODH, Dihydroorotate Dehydrogenase; OA, orotate; ACSL4, acyl-CoA synthetase long-chain family member 4; LPCAT3, lysophosphatidylcholine acyltransferase 3; ALOXs, arachidonate lipoxygenases; POR, cytochrome P450 oxidoreductase.*

Ferroptosis is regulated by the iron metabolism, lipid metabolism, and antioxidant systems (Figure 1). Acyl-CoA synthetase long-chain family member 4 (ACSL4) and lysophosphatidylcholine acyltransferase 3 (LPCAT3) are the critical mediators of polyunsaturated phospholipid (PUFA-PL) synthesis. ACSL4 catalyzes the binding of free polyunsaturated fatty acids (PUFAs) to CoA to generate PUFA-CoA (Doll et al., 2017), which is then esterified by LPCAT3 and linked to phospholipids to form PUFA-PLs (Dixon et al., 2015). PUFA-PLs can be oxidized by cytochrome P450 oxidoreductase (POR) and arachidonate lipoxygenases (ALOXs) to produce lipid peroxides (L-OOH) (Dixon et al., 2015; Shintoku et al., 2017; Zou et al., 2020b). Typically, L-OOH is reduced to the corresponding alcohols by GPX4 (Yang et al., 2014). However, a large amount of L-OOH reacts with Fe^2+^ to produce hydroxyl radicals (L-OO^.^) when Fe^2+^ and PUFAs are overloaded, or the expression of glutathione peroxidase (GPX4) and glutathione (GSH) is reduced. Accumulation of l-OO^.^ results in lipid peroxidation of membrane phospholipids, ultimately leading to ferroptosis (Dixon et al., 2012). Recently, ferroptosis was observed in many types of cancer, such as head and neck (Tang et al., 2021), breast (Zhang et al., 2021b), and lung cancers (Ren et al., 2021). Ferroptosis can function in cancer progression and suppression by regulating gene expression and the tumor microenvironment (TME) (Wang et al., 2020c).

*P53* is one of the tumor suppressor genes that play a critical role in tumor suppression. It can reportedly regulate genes involved in ferroptosis to suppress tumor development. *P53* can downregulate the expression of cystine/glutamate antiporter solute carrier family seven member 11 (SLC7A11), a critical factor in glutathione synthesis, to inhibit GSH synthesis (Jiang et al., 2015). *P53* can also induce ferroptosis by promoting the expression of arachidonate 15-lipoxygenase (ALOX15) via its transcriptional target spermidine/spermine N1-Acetyltransferase 1 (SAT1) (Ou et al., 2016). In addition, it can act on other enzymes that control phospholipid and iron contents, such as prostaglandin-endoperoxide synthase 2 (PTGS2) and ferredoxin reductase (FDXR), to promote ROS production (Yang et al., 2014; Zhang et al., 2017). However, *P53* can also inhibit ferroptosis. Dipeptidyl peptidase 4 (DPP4) is a regulatory molecule involved in ferroptosis and lipid metabolism. It binds to nicotinamide adenine dinucleotide phosphate oxidase 1 (NOX1) to mediate ROS production. *P53* can reduce DDP4 levels and block its binding with NOX1 to inhibit ferroptosis (Xie et al., 2017). Besides, *P53* can activate cyclin-dependent kinase inhibitor 1 (CDKN1A)/p21 to increase GSH content, thus inhibiting ferroptosis (Tarangelo et al., 2018). The regulation of *P53* in ferroptosis seems to be highly dependent on the environment, although the exact mechanism remains unknown (Kang et al., 2019; Liu et al., 2020). *BAP1*, another tumor suppressor gene, has also been linked to ferroptosis. *BAP1* can reduce histone 2A ubiquitination (H2Aub) on chromatin to suppress the expression of SLC7A11, thus inducing ferroptosis (Zhang et al., 2018).

The TME refers to the internal environment closely related to tumor generation and progression. According to several researches, ferroptosis plays a crucial role in controlling immune cell function. On the one hand, the ferroptosis of immune cells reduce their number and function, impairing the immune response. On the other side, the ferroptosis of non-immune cells will cause the release of damage-associated molecular pattern (DAMP) and trigger immune responses (Wang and Lu, 2022). Zou et al. (Wang et al., 2019) found that IFN γ derived from immunotherapy-activated CD8^+^ T cells could down-regulate SLC3A2 and SLC7A11 levels, leading to decreased cystine uptake and enhanced tumor lipid oxidation and ferroptosis. Besides, radiation can also inhibit the expression of SLC7A11, synergistic with CD8^+^ T cells to improve tumor control (Lang et al., 2019). Thus, ferroptosis may be the focus for the development of effective combinatorial cancer therapy. In addition, ferroptosis drives the polarization of macrophages in the TME. Autophagy-dependent ferroptosis (ADF) enables the release of the KRAS^G12D^ protein from cancer cells into the TME, and KRAS^G12D^ induces the transformation of macrophages into an M2 phenotype, accelerating cancer development (Dai et al., 2020). However, Hsieh et al. found that Zero-valent-iron (ZVI) nanoparticle (NP) which functions like a ferroptosis inducer could efficiently repolarizes macrophages from M2 phenotype to M1 phenotype (Hsieh et al., 2021). Therefore, the relationship between ferroptosis and macrophage repolarization still deserves further discussion. Ferroptosis is also involved in the treatment of B lymphocytes immune-related diseases, such as immune deficiency disease and diffuse large B-cell lymphoma (DLBCL) (Criscitiello et al., 2020; Schmitt et al., 2021), but there is insufficient evidence showing a link between ferroptosis and tumor-infiltrating B lymphocytes. What’s more, hypoxia, a well-known feature of the TME, is also involved in ferroptosis (Su et al., 2022). Hypoxia-inducible factor (HIF)-1α can prevent ferroptosis by inhibiting the expression of SLC7A11 (Jiang et al., 2017; Fan et al., 2021). However, HIF-2α can stimulate the expression of hypoxia-induced lipid droplet-associated proteins (HILPDA) and enrich the amount of polyunsaturated lipids in cells to increase their sensitivity to ferroptosis (Zou et al., 2019). In general, ferroptosis plays a vital role in the development and progression of malignancies. Additional research into its mechanisms may lead to new cancer therapy options.

## 3 Radiation induced ferroptosis in tumor cells

**FIGURE 2:**
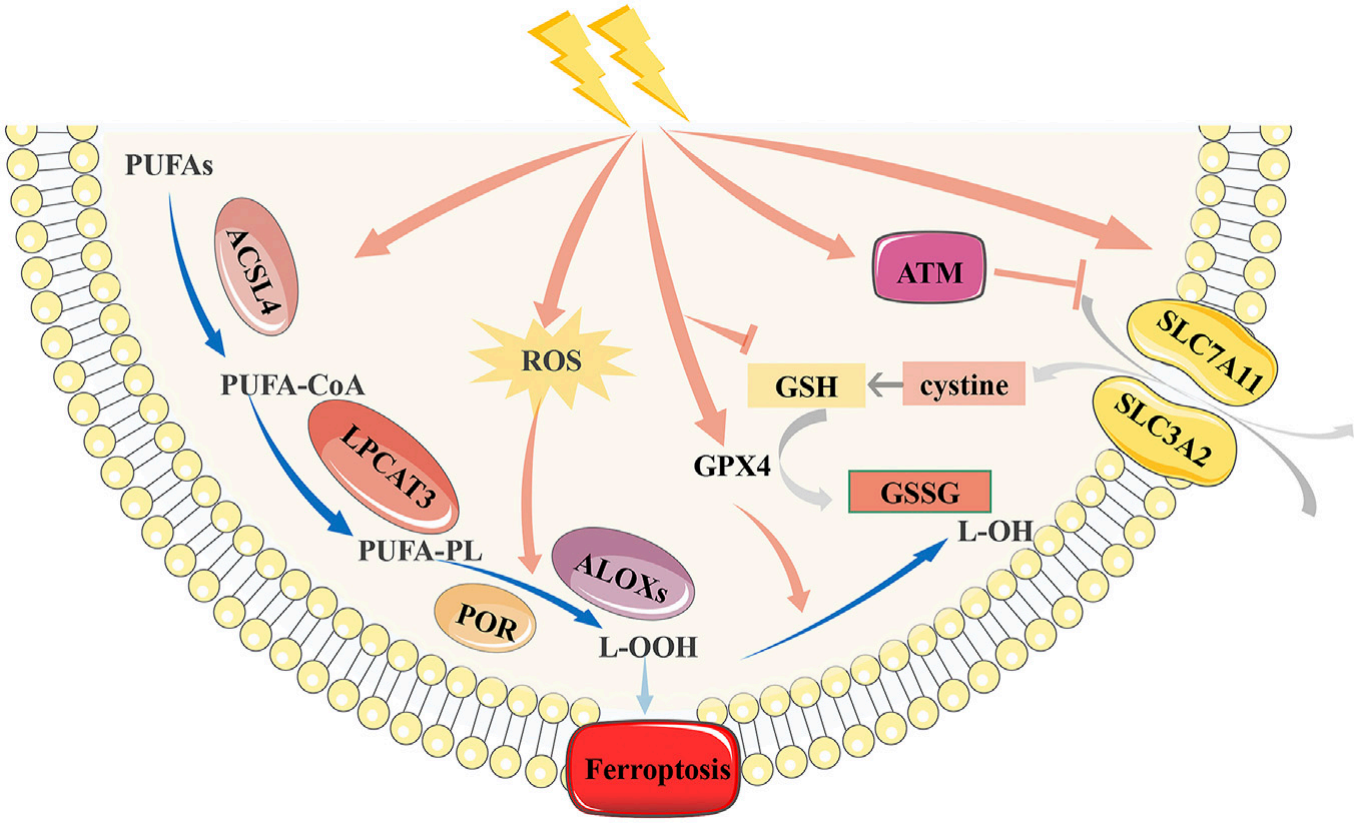
Mechanism of ferroptosis induced by radiotherapy. Radiation can inhibit the antioxidant function of SLC7A11 and GSH, and up-regulate the expression of ACSL4, thus inducing ferroptosis. However, radiation can aslo up-regulate the expression of SLC7A11 and GPX4 due to adaptive response. *SLC7A11, Cystine/glutamate antiporter solute carrier family seven member 11; GPX4, glutathione peroxidase 4; GSH, glutathione; ACSL4, acyl-CoA synthetase long-chain family member 4.*

Radiation was assumed to primarily cause apoptosis; however, new studies have discovered a large amount of evidence demonstrating a strong link between radiation and ferroptosis (Lang et al., 2019; Lei et al., 2020; Ye et al., 2020) (Figure 2). Lang et al. (2019) observed that IR-induced ferroptosis is mainly associated with lipid peroxidation in tumor cells. They found that irradiation can activate the ataxia-telangiectasia mutated gene (*ATM*), inhibit the expression of SLC7A11, and cause lipid peroxidation, thereby inducing ferroptosis. Lei et al. (2020) subsequently verified this conclusion and suggested that IR significantly increases ACSL4 expression in cancer cells, with typical morphological changes in ferroptosis. However, they found that IR induced a significant upregulation of ferroptosis suppressor genes, including *SLC7A11* and *GPX4*, which may be an adaptive response. Ye et al. (2020) further refined the mechanism of IR-induced ferroptosis. GSH is a vital regulator of the antioxidant system, which can convert H_2_O_2_ to H_2_O under the action of GPX4, thus protecting cells from external stimuli (Brigelius-Flohé, 2006). IR can consume GSH by generating a large number of ROS and limit the synthesis of GSH by inhibiting cystine uptake, finally leading to GSH depletion. And GSH shortage will impair the activity of the cellular antioxidant system and cause ferroptosis.

In addition to killing tumor cells via ROS, IR can directly damage cellular DNA and produce double-strand breaks. Various lines of evidence imply that IR-induced ferroptosis has little effect on DNA damage, whereas IR-induced DNA damage appears to affect ferroptosis through diverse mechanisms (Lei et al., 2021). Many DNA damage response (DDR) components regulate ferroptosis. For example, *ATM* activation promotes ferroptosis through the metal regulatory transcription factor 1 (MTF1) -Ferritin/FPN1 axis and SLC7A11-GSH pathway (Lang et al., 2019; Chen et al., 2020). *P53* also regulates ferroptosis by modulating DPP4, SAT1, and other factors (Ou et al., 2016; Xie et al., 2017). However, these ferroptosis-regulating target genes are not directly involved in the canonical phenotypic effects of DDR; most of them affect ferroptosis using noncanonical mechanisms. Therefore, it is reasonable to infer that ferroptosis represents a back-up death method of canonical cell death for cells with DNA damage. Further characterization of their interactions may generate new insights into tumor suppression.

To sum up, IR-induced ferroptosis is regulated by four pathways. First, IR promotes ROS production and upregulates ACSL4 expression. IR regulates the expression of SLC7A11 according to the external environment. Moreover, IR directly leads to GSH depletion and the destruction of antioxidant systems. Besides, IR-induced DNA damage also regulates the occurrence of ferroptosis. Ferroptosis accounts for a large proportion of radiation-induced cell death cases. This discovery facilitates the application of ferroptosis inducers (FINs) in radiotherapy.

## 4 Inducing ferroptosis enhances radiosensitivity

With the increasing application of radiotherapy, improving the radiosensitivity of tumor cells to increase its effectiveness has become an increasingly important issue. The iron, lipid, and antioxidant systems involved in regulating ferroptosis can adjust radiosensitivity (Zhang and Martin, 2014; Theriot et al., 2016; Nisticò et al., 2021). Therefore, the relationship between ferroptosis and radiosensitivity is worth investigating.

### 4.1 Iron metabolism and radiosensitivity

Iron is a vital component of the human body and is linked to metabolism, cell death, and the development of various diseases. Deferoxamine (DFO) was used to eliminate FIN-induced cell death caused by FINs, implying that iron is a vital element in ferroptosis (Dixon et al., 2012). In the human body, iron absorption mainly occurs in the intestine, where Fe^2+^ can be oxidized by ceruloplasmin to Fe^3+^ and binds to transferrin (TF) on the cell membrane to form TF-Fe^3+^. TF-Fe^3+^ can enter cells by integrating with TF receptor 1 (TFR1) (Frazer and Anderson, 2014). Fe^3+^ is then reduced to Fe^2+^ by six transmembrane epithelial antigens of prostate 3 (STEAP3) and stored in the labile iron pool and ferritin (Bogdan et al., 2016). When Fe^2+^ is overloaded, ferroptosis is induced.

In addition, iron homeostasis plays a vital role in radiotherapy. Rapidly dividing tumor cells require much more iron than normal cells; therefore, iron deprivation is a new cancer treatment option. The iron-chelating agent can reduce free Fe^2+^ content and arrest cells at the G2/M phase, thereby enhancing the radiosensitivity of cancer cells (Turner et al., 2005). In addition, Mir-7-5p could lead to ferroptosis resistance by downregulating mitoferrin and reducing Fe^2+^ content. The application of an Mir-7-5p inhibitor can reverse this effect and improve radiosensitivity (Tomita et al., 2019).

Thus, iron homeostasis serves as a link between ferroptosis and RT. Other iron-metabolizing proteins, such as heat shock protein B1 (HSPB1) (Chen et al., 2006) and iron response element-binding protein 2 (IREB2) (Mumbauer et al., 2019; Li et al., 2020), may also be potential targets for enhancing radiosensitivity.

### 4.2 Lipid metabolism and radiosensitivity

There is growing evidence that lipid metabolism, ferroptosis, and radiation are inextricably linked. Ferroptosis is mainly caused by the peroxidation of PUFA-PLs (Yang and Stockwell, 2016). Previously, this process was thought to be primarily catalyzed by arachidonate lipoxygenases (ALOXs). However, recent studies have shown that cytochrome P450 oxidoreductase (POR) plays a more significant role (Yan et al., 2021). Radiation can also activate ALOXs and POR to mediate lipid peroxidation (Wei et al., 2019). The radiotherapeutic efficacy in head and neck cancer has been proven to be enhanced by 15-LOX overexpression (Yang et al., 2008). However, 12-LOX has been linked to radioresistance in prostate cancer cells (Lövey et al., 2013). ACSL4 is a crucial protein involved in the formation of PUFA-PLs. ACSL4 knockout cells show superior resistance to ferroptosis (Doll et al., 2017). However, ACSL4 also negatively affects radiosensitivity in breast cancer by regulating forkhead box M1 (FOXM1) to enhance the DNA damage response and inhibit apoptosis (Kwon et al., 2021). These results demonstrated that enzymes related to phospholipids play a complex role in radiation and ferroptosis. This phenomenon is possible because they also participate in radiation-induced inflammatory responses (Kim et al., 2018). More research into these enzymes and their lipid metabolites will help us better understand the link between radiation and ferroptosis, allowing us to improve the efficacy of radiotherapy.

**FIGURE 3:**
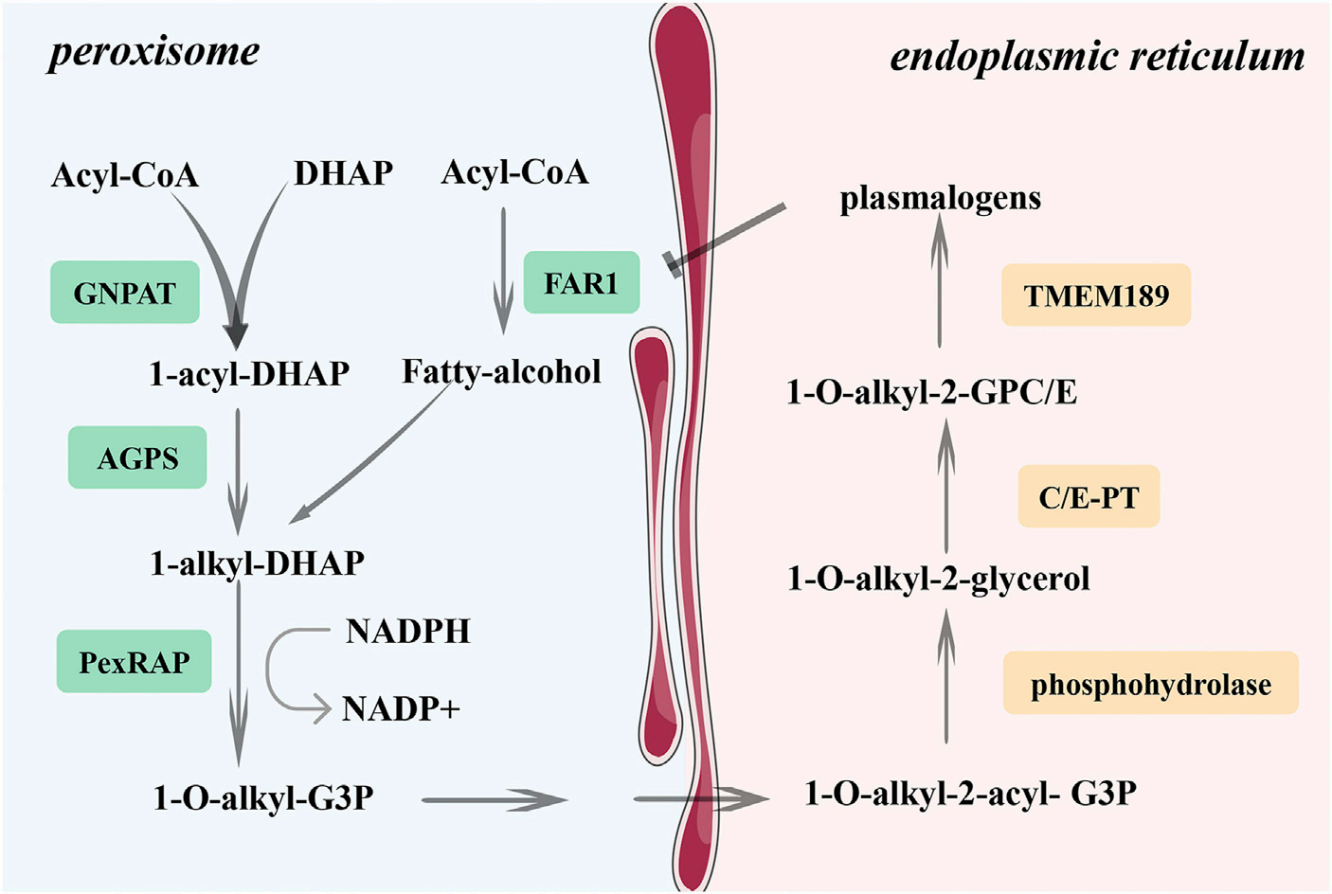
Synthesis of plasmalogens. FAR1 is a key enzyme in the synthesis of plasmalogen. It can convert saturated fatty alcohols into unsaturated fatty. The *TMEM189* gene encodes plasmanylethanolamine desaturase, which could introduce the vinyl ether double bond into plasmalogens and convert alkyl-ether lipids into plasmalogens. However, FAR1 is regulated by the negative feedback of phospholipid level, and it can be inhibited by an increase in plasmalogens content. *FAR1, fatty acyl-CoA reductase 1; GNPAT, glyceronephosphate-O-acyltransferase; AGPS, alkylglycerone phosphate synthase.*

In addition, peroxisomes participate in ferroptosis by synthesizing ether phospholipids (Zou et al., 2020a). Ether phospholipids represent a group of phospholipids containing fatty alcohol at the stereospecific numbering (sn)-1 position. Plasmalogens appeared to be the most abundant. Its production is regulated by the fatty acyl-CoA reductase 1 (FAR1)–transmembrane 189 (TMEM189) axis (Cui et al., 2021) (Figure 3). FAR1 converts saturated fatty alcohols to unsaturated fatty alcohols and promotes the formation of alkyl ether lipids. The *TMEM189* gene encodes plasmanylethanolamine desaturase, introducing a vinyl ether double bond into alkyl ether lipids and converting alkyl-ether lipids into plasmalogens (Werner et al., 2020). Zou et al. (2020a) demonstrated that peroxisome-mediated ferroptosis is only related to unsaturated fatty acids on ether lipids but not alkyl and vinyl groups. Cui et al. (2021) suggested that vinyl groups in plasmalogens are at least partly responsible for the prevention of ferroptosis. The regulation of ferroptosis by ether glycerophospholipids is related to their content and cell localization (Balgoma and Hedeland, 2021). As a result, the differences in the above two outcomes may be caused by the different ratios of alkyl-ether phospholipids to plasmalogens in different cells. As alkyl phospholipid ether analogs have been proven to enhance the radiosensitivity of solid tumors (Elsaid et al., 2018), further research on the FAR1-TMEM189 pathway may provide new targets for radiosensitization.

### 4.3 Antioxidant system and radiosensitivity

Ferroptosis is strongly related to oxidative stress, and there are numerous overlapping regulation pathways. Antioxidants like superoxide dismutase (SOD), GSH, and NADPH can prevent tumor cell death by inhibiting oxidative stress. Studies have shown that ferroptosis is regulated by antioxidant systems as well. Four pathways maintain redox homeostasis during ferroptosis: the GSH/GPX4, FSP1-CoQ10-NAD (P)H, GCH1-BH4-DHFR, and DHO-DHODH-OA pathways (Bersuker et al., 2019; Doll et al., 2019; Mao et al., 2021). The radiosensitivity of tumor cells can be enhanced by adjusting these regulatory pathways (Lei et al., 2020).

#### 4.3.1 SLC7A11-GSH-GPX4 pathway

Together, cystine uptake, GSH biosynthesis, and GPX4 activation constitute a robust defense system that keeps lipid hydroperoxides below toxicity thresholds to prevent ferroptosis. SLC7A11 (also known as xCT) is a functional subunit of system X_c__,_^−^ which can exchange extracellular cystine with intracellular glutamate in a 1:1 ratio (Bannai, 1986). When cystine enters the cell, it is reduced to cysteine, which is the rate-limiting precursor of glutathione synthesis. GPX4 uses GSH as a cofactor to reduce PL-OOH to nontoxic PL-alcohols, thereby inhibiting ferroptosis. In addition, GPX4 maturation requires selenium. The maturation of selenocysteine tRNA is controlled by the mevalonate (MVA) pathway (Warner et al., 2000). Therefore, the regulation of the MVA pathway can also control ferroptosis.

Erastin is a classic FIN that can directly inhibit system X_c_^−^ and thus decrease the level of glutathione (Dixon et al., 2012). Erastin increases the radiosensitivity of lung cancer cells by inducing ferroptosis (Pan et al., 2019). Studies have also confirmed that erastin can achieve radiosensitizing effects by consuming GSH and reducing antioxidant capacity *in vivo* (Shibata et al., 2019). In addition, FINs targeting GPX4, such as RSL3 and ML162, can enhance the radiosensitivity of tumor cells (Lei et al., 2020). Thus, inhibition of the SLC7A11-GSH-GPX4 pathway can achieve radiosensitization by promoting ferroptosis, and SLCA11, GPX4, and GSH should serve as targets for radiosensitization.

The genes upstream of *SLC7A11* can also regulate radiosensitivity. Nuclear factor erythroid 2-related factor 2 (*Nrf-2*) and activating transcription factor 4 (*ATF4*) can promote *SLC7A11* expression at the transcriptional level (Lewerenz and Maher, 2009). Hyperactivation of Nrf-2 leads to radiation resistance by inducing SLC7A11 expression and reducing lipid peroxidation levels *in vitro* (Feng et al., 2021a). Furthermore, RNA-binding proteins participate in the regulation of ferroptosis. Recently, the RNA-binding motif, single-stranded-interacting protein 1 (RBMS1), an RNA-binding protein, was found to directly interact with the translation initiation factor eIF3d, connecting the 3′- and 5′-UTRs of *SLC7A11*. RBMS1 ablation can sensitize radioresistant lung cancer cells to radiation by inhibiting SLC7A11 translation and inducing ferroptosis (Zhang et al., 2021c).

In addition to SLC7A11, other factors that regulate GPX4 and GSH levels can be radiation targets. 5-aminolevulinic acid (5-ALA) is a natural amino acid widely used in cancer treatment. Its radiosensitizing effect has been reported in various cancers (Yamamoto et al., 2012; Yamada et al., 2019; Tung et al., 2021). A previous study confirmed that 5-ALA promotes the synthesis of GPX4 and induces ferroptosis (Shishido et al., 2021). Transmembrane protein 27 (CLTRN, also known as TMEM27) is a type Ia transmembrane (Akpinar et al., 2005), which can be regulated by the Nrf-1/RAN/DLD protein complex to deplete GSH and enhance the radiosensitivity of hepatocellular carcinoma (HCC) cells (Yuan et al., 2021). Therefore, the SLC7A11-GSH-GPX4 pathway regulates radiosensitization, which may be related to ferroptosis.

#### 4.3.2 Ferroptosis suppressor protein 1-CoQ10-NAD (P)H pathway

Ferroptosis suppressor protein 1 (FSP1) [also known as apoptosis-inducing factor mitochondrial 2 (AIFM2)] is a potent ferroptosis suppressor. According to one study, the FSP1-CoQ(10)-NAD(P)H pathway exists as a separate parallel system that, in conjunction with GPX4, inhibits phospholipid peroxidation and ferroptosis. FSP1 is recruited to the plasma membrane by myristoylation, where it reduces coenzyme Q10 (CoQ) to ubiquinol (CoQH2) with the help of NAD(P)H. CoQH2 then suppresses ferroptosis by capturing lipophilic free radicals (Bersuker et al., 2019; Doll et al., 2019). As a result, CoQH2 production is critical for the proper operation of the FSP1-CoQ(10)-NAD(P)H pathway.

FIN56, a special ferroptosis inducer, can bind and activate squalene synthase, leading to depletion of the endogenous antioxidant CoQ10 (Shimada et al., 2016). FIN56 can induce lipid peroxidation and significantly radiosensitize lung cancer cells (Lei et al., 2020). Statins are lipid-lowering drugs that induce ferroptosis. It can inhibit GPX4 by regulating selenoprotein and CoQ biosynthesis through the MVA pathway (Warner et al., 2000; Santoro, 2020). Statins have been confirmed to act as radiation sensitizers in various tumor cells (Hutchinson and Marignol, 2017; Jin et al., 2018; Aschenbrenner et al., 2021). In general, the FSP1-CoQ (10)-NAD(P)H pathway is closely related to radiosensitivity, with CoQ being the most critical target.

#### 4.3.3 GTP-cyclohydrolase-1-Tetrahydrobiopterin-dihydrofolate reductase pathway

Tetrahydrobiopterin (BH4) is a redox-active cofactor that boosts CoQH2 production (Crabtree et al., 2009). The formation of BH4 and BH2 is induced by the expression of GTP-cyclohydrolase-1 (GCH1), and BH2 can be reduced to BH4 by dihydrofolate reductase (DHFR). GCH1 is the primary enzyme involved in this process. Kraft et al. 2020 observed that GCH1 overexpression protects cells against ferroptosis triggered by FINs, such as RSL3, IKE, and GPX4. The GCH1-BH4-phospholipid axis controls the endogenous production of BH4, the abundance of CoQ10, and the depletion of unusual phospholipids with two polyunsaturated fatty acyl tails. Soula et al. (2020) demonstrated that tumor cells are sensitive to RSL3, which can directly inactivate GPX4 when GCH1 is deleted or inhibited. Furthermore, inhibiting the action of DHFR with methotrexate can also increase the sensitivity of cells to ferroptosis. These findings imply that the GCH1-BH4-DHFR route, as an endogenous antioxidant pathway, inhibits ferroptosis via a mechanism unrelated to the GPX4 system.

GCH1 and DHFR are highly expressed in various cancers, and radiation can further activate BH4 metabolic enzymes to reduce radiotherapy effects, proving that BH4 metabolic enzymes are possible hallmarks and targets of radiosensitivity (Yan et al., 2020; Feng et al., 2021b). Liang et al. (2020) synthesized a series of 2,4-diaminopteridine analogs as DHFR inhibitors and demonstrated that inhibiting DHFR improves the irradiation effect on cervical cancer cells.

#### 4.3.4 Dihydroorotate dehydrogenase-orotate pathway

Dihydroorotate dehydrogenase (DHODH) is a flavin-dependent enzyme found primarily on the inner membrane of mitochondria and is required for *de novo* pyrimidine nucleotide synthesis. It can catalyze the conversion of dihydroorotate (DHO) to orotate (OA) (Femia et al., 2021). Mao et al. (2021) found that, while DHODH oxidized DHO to OA, CoQ was reduced to CoQH2, thus inhibiting ferroptosis. DHODH works in tandem with mitochondrial GPX4 but is unaffected by cytosolic GPX4 or FSP1. Mechanistically, DHODH eliminates L-OO^.^ through CoQH2, thus playing a synergistic role with GPX4 in inhibiting ferroptosis (Femia et al., 2021).

Pharmacological inhibition of DHODH can effectively reduce the viability of small-cell lung cancer cells (Li et al., 2019a). The relationship between DHODH and radiosensitivity remains unclear. However, ultraviolet-B (UVB) can activate DHODH, and DHODH inhibition can decrease DNA repair ability (Hosseini et al., 2019). This result proves that the relationship between DHODH and radiosensitivity is worth investigating.

Radiation can generate superfluous ROS, which destroys proteins, DNA, lipids, and other biological macromolecules in cells, leading to cell death (Wang et al., 2020b). The radiosensitivity of tumor cells can be improved by increasing ROS levels and promoting oxidative stress. However, excessive ROS can activate the antioxidant system and render radiation-resistant tumor cells. Ferroptosis is accompanied by reactive oxygen species (ROS) overload and redox imbalance.

## 5 Inhibition of ferroptosis can reduce radiation damage

Despite ongoing advancements in radiation technology, radiation damage to normal tissues is unavoidable (Liu et al., 2021). Overcoming the side effects of radiotherapy remains a hot research topic. Radiation can induce ferroptosis in both tumor and normal cells. Ferroptosis has been reported to play a role in the radiation-induced lung, intestinal, skin, and hematopoietic injuries. Inhibition of ferroptosis may be an effective way to alleviate radiation injury (Table 2).

**TABLE 2:** Targets and mechanisms of ferroptosis inhibitors alleviating radiation injury.

Damage Type	Drugs	Target	Mechanism	References
Radiation-induced lung injury	Liproxstatin-1	GPX4	Increase GPX4 levels	(Li et al., 2019b)
	GsMTx4	PIEZO1	Inhibit PIEZO1/Ca2+/calpain pathway and increase the expression GPX4 and SLC7A11	Guo et al. (2021)
	PD151746	calpain	Inbibit calpain and increase the expression GPX4 and SLC7A11	Guo et al. (2021)
	NVP-AUY922	HSP90	Inhibit HSP90/CMA pathway and inhibit the degradation of GPX4	(Li et al., 2022)
Radiation-induced intestinal injury	Liproxstatin-1	LPCAT3	Inhibit radiation-activated LPCAT3/LOX pathway	Wang et al. (2022)
	(-)-epigallocatechin-3-gallate	Nrf-2	Increase the expression of GPX4 and SLC7A11 and reduce ROS content by activating Nrf-2	Xie et al. (2020)
Radiation-induced skin injury	NMN	NAD+	Promote the NAD+/NADH system, increase the synthesis of GSH, and enhance the resistance to ferroptosis in a GPX4-dependent manner	Feng et al. (2022)
Radiation-induced hematopoietic injury	Ferrostatin-1	GPX4	Reduce the levels of hemosiderin and liable iron pool and increase GPX4	(Zhang et al., 2021d)
Total body irradiation induced injury	Baicalein	15-LOX	Inhibit depletion of GPX4 and 15-LOX	Dar et al. (2022)
	Polycysteine	GPX4，NOX1	Activate GPX4 and inhibit NOX1	Zhang et al. (2021a)

GPX4, glutathione peroxidase 4; HSP90, the heat shock protein 90; CAM, chaperone-mediated autophagy; LPCAT3, lysophosphatidylcholine acyltransferase 3; NMN, nicotinamide mononucleotide; LOX, lipoxygenase; Nrf-2, Nuclear factor erythroid 2-related factor 2; GSH, glutathione; NOX1, nicotinamide adenine dinucleotide phosphate oxidase.

### 5.1 Radiation-induced lung injury

Radiation-induced lung injury (RILI) is one of the most common and serious complications of radiotherapy for thoracic malignancies (S et al., 2019). This process is often accompanied by the upregulation of inflammatory cytokines such as interleukin-6, 10, and transforming growth factor-β1 (Szabo et al., 2010). ROS are also believed to be a key factor, and their accumulation is the basis of ferroptosis (Yin et al., 2019; Li et al., 2020). An increasing number of studies have investigated the association between ferroptosis and RILI.

**FIGURE 4:**
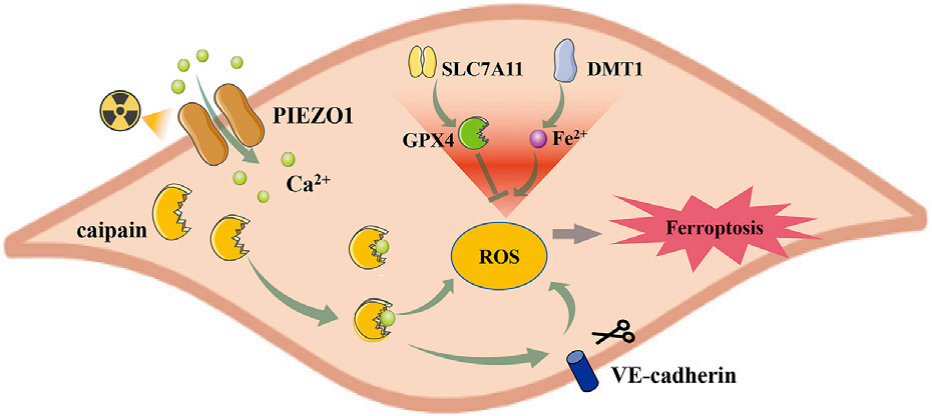
PIEZO1 regulates radiation-induced ferroptosis in lung endothelial cells. Radiation increased the expression of PIEZO1 in the lung endothelial cells, which caused the influx of Ca^2+^. Increased intracellular Ca^2+^ activates caipain and promotes ferroptosis by regulating SLC7A11, GPX4, and DMT1 expression. Besides, the Ca2+/Caipain pathway can also promote the degradation of VE-cadherin, leading to ferroptosis. *PIEZ O 1, Piezo-type mechanosensitive ion channel component 1; SLC7A11, Cystine/glutamate antiporter solute carrier family seven member 11; GPX4, glutathione peroxidase 4; DMT1, divalent metal transporter 1.*

In acute RILI, ferroptosis characteristics of mitochondria have been detected, together with substantial downregulation of GPX4 levels (Li et al., 2019b; Guo et al., 2021). Piezo-type mechanosensitive ion channel component 1 (PIEZO1) is a mechanically activated calcium channel highly expressed in lung tissue (Coste et al., 2010). PIEZO1/Ca^2+^/calpain signaling mediates radiation-induced ferroptosis in pulmonary endothelial cells (Guo et al., 2021) (Figure 4). Radiation induces PIEZO1 protein overexpression, elevating intracellular Ca^2+^ levels and activating the Ca^2+^/calpain signaling pathway. Calpain then lowered the expression of GPX4 and SLC7A11, whereas it increased the expression of divalent metal transporter 1 (DMT1), which is responsible for iron uptake, induces ferroptosis, and promotes RILI. Vascular endothelial cadherin (VE-cadherin) is a calcium-dependent adhesive molecule expressed only in endothelial cells (Gory et al., 1999). Activation of the Ca^2+^/calpain signaling pathway can promote its degradation, leading to increased ROS content and ferroptosis (Guo et al., 2021). However, the mechanism underlying VE-cadherin-induced ferroptosis has not been comprehensively investigated.

Correspondingly, inhibition of ferroptosis can alleviate RILI. Liproxstatin-1 (Lip-1), a ferroptosis inhibitor, can reduce characteristic ferroptotic changes, increase GPX4 levels, and alleviate symptoms of RILI, such as lung injury and hemorrhage. In addition, it can reduce the levels of inflammatory factors, suggesting that ferroptosis plays a role in the inflammatory microenvironment (Li et al., 2019b). Chaperone-mediated autophagy (CMA) is usually activated in response to stress, and its activation can cause the degradation of GPX4 (Yu et al., 2022). The heat shock protein 90 (HSP90) is crucial in activating CMA (Bandyopadhyay and Cuervo, 2008). NVP-AUY922, an HSP90 inhibitor, can alleviate RILI by inhibiting autophagy-dependent ferroptosis (Li et al., 2022). Therefore, ferroptosis is closely related to RILI, and ferroptosis inhibitors may play a role in RILI prevention and treatment.

### 5.2 Radiation-induced intestinal injuries

Radiation-induced intestinal injury (RIID) is a common complication of RT for pelvic malignancies. The clinical symptoms caused by RIID seriously affect the quality of life and even lead to death (MacNaughton, 2000). Ferroptosis regulates intestinal immune function and the occurrence of RIID (Wang et al., 2022). Radiation can cause a reduction in intraepithelial lymphocyte (IELs) count and overexpression of interferon-gamma, transforming growth factor, and other immune system changes. This effect was suppressed by Lip-1 treatment. The regulatory effect of ferroptosis on intestinal immune function may be related to the radiation-activated LPCAT3/LOX pathway (Wang et al., 2022). Epigallocatechin-3-gallate (EGCG) is a major polyphenol with a potent antioxidant activity (Zhong et al., 2012). Xie et al. (2020) observed that EGCG alleviated RIID by increasing the expression of GPX4 and SLC7A11 and inhibiting ferroptosis after radiotherapy. Therefore, regulating the ferroptosis pathway to prevent and treat RIID provides a new strategy for radiation protection.

### 5.3 Radiation-induced skin injury

As a result of radiotherapy, approximately 85%–95% of patients experience varying degrees of skin damage (Yang et al., 2020). Radiation-induced skin injury (RISIs) consists of both acute and chronic injuries. Acute injuries include erythema, dry and wet desquamation, edema, bleeding, and ulcers. The chronic injury involves keratosis, telangiectasias, fibrosis, and skin cancer (Hymes et al., 2006, y). Radiation-induced ROS production is closely related to RISIs, and common ROS regulatory pathways, such as the Keap1/Nrf-2 and GCH1/BH4 pathways, play a seminal role in preventing RISIs (McNeill et al., 2015; Xue et al., 2017; Wei et al., 2021). As a form of cell death caused by ROS overload, ferroptosis is also involved in RSIs. Feng et al. (Feng et al., 2022) discovered that ultraviolet (UV) exposure could induce the accumulation of lipid peroxides in human skin keratinocytes and cause intracellular Fe^2+^ overload by regulating the levels of TFRC, FTL, FTH, and the iron exporter ferroportin (FPN). Nicotinamide mononucleotide (NMN) is the precursor of NAD^+^, which significantly promotes the NAD^+^/NADH system, increases the synthesis of GSH, and enhances resistance to ferroptosis in a GPX4-dependent manner. The application of NMN can significantly reduce RISIs. Therefore, ferroptosis inhibitors may be an effective therapeutic approach for treating irradiation-induced skin damage. However, whether this conclusion applies to radiotherapy-induced damage remains unclear.

### 5.4 Radiation-induced hematopoietic injury

Radiation can inhibit the division and proliferation of hematopoietic cells in the bone marrow, causing a decline in peripheral blood images and increasing the risk of secondary infection. Studies have shown that radiation can cause ferroptosis in bone marrow mononuclear cells (BMMCs) by increasing iron and lipid peroxidation levels and depleting GPX4 and GSH. Ferrostatin-1, a ferroptosis inhibitor, can mitigate the ferroptosis of BMMCs and increase the number of red blood cells, white blood cells, lymphocytes, and monocytes in the peripheral blood of irradiated mice (Zhang et al., 2021d). Another study showed that radiation-induced hemorrhage could induce ferroptosis in granulocyte-macrophage hematopoietic progenitor cells, and anti-ferroptosis can ameliorate hematopoietic injury (Zhang et al., 2020). Although the exact mechanism remains unknown, blocking ferroptosis can help reduce radiation-induced hematopoietic injury.

Inhibition of ferroptosis also reduced the mortality caused by radiation in mice. It was proved that *Pseudomonas aeruginosa* (PAO1) could stimulate ferroptosis by making PAO1 15-lipoxygenase (pLoxA) act on 15-LOX and GPX4 and significantly reduce the survival of irradiated mice. The application of baicalein, a lipoxygenase inhibitor, reversed this phenomenon and reduced mortality in mice exposed to total body irradiation (TBI) and PAO1 infection (Dar et al., 2022). Polycysteine can improve the survival of mice exposed to TBI and reduce radiation-induced damage by activating GPX4 and inhibiting nicotinamide adenine dinucleotide phosphate oxidase 1 (NOX1) (Zhang et al., 2021a).

The radiation-induced buildup of ROS causes oxidative stress in normal cells (Shen et al., 2018; Yahyapour et al., 2018). Ferroptosis, caused by ROS overload, is closely related to oxidative stress. Radiation injury is often accompanied by an inflammatory response. Ferroptosis is often associated with inflammatory manifestations by regulating the activity of LOXs and PTGS2 (Sun et al., 2020). Therefore, the role of ferroptosis in radiation-induced injury is essential. Radiation-mediated ferroptosis can damage normal tissues by overloading ROS and Fe^2+^, disrupting the antioxidant systems, and inducing inflammatory responses. Its functions have been verified in multiple radiation-induced system injuries. However, it is unclear if these mechanisms apply to all systems and whether various systems have distinct specialized targets.

## 6 Conclusion and future perspectives

Ferroptosis, as a mode of cell death receives increasing attention, has shown great potential for cancer treatment. IR can induce ferroptosis by producing ROS, upregulating the expression of ASCL4, depleting GSH, and promoting lipid peroxidation. Besides, many DDR pathway components can also be activated by IR to affect ferroptosis through noncanonical mechanisms. Similarly, stimulating ferroptosis by regulating iron metabolism, lipid metabolism, and antioxidant systems can enhance the radiosensitivity of tumor cells, providing a new technique for increasing the efficacy of radiotherapy. FINs have not been widely used in clinical practice owing to their high toxicity, inadequate targeting, and other factors. To satisfy the therapeutic needs, new FINs that are more effective and stable should be created *in vivo*. In addition, ferroptosis is implicated in radiation-induced normal tissue damage by increasing ROS and Fe^2+^ levels, altering antioxidant systems, and generating inflammatory responses. Therefore, the question of how to promote ferroptosis in tumor cells while sparing normal cells remains unresolved.
